# Clinical efficacy of local injection therapies for lateral epicondylitis: A systematic review and network meta-analysis

**DOI:** 10.22088/cjim.13.2.1

**Published:** 2022

**Authors:** Mehdi Tavassoli, Rahmatollah Jokar, Mohammad Zamani, Soraya Khafri, Seyed Mokhtar Esmaeilnejad-Ganji

**Affiliations:** 1 Clinical Research Development Center, Shahid Beheshti Hospital, Babol University of Medical Sciences, Babol, Iran; 2 Department of Orthopedics, Babol University of Medical Sciences, Babol, Iran; 3 Student Research Committee, Babol University of Medical Sciences, Babol, Iran; 4 Biostatistics and Epidemiology Department, Faculty of Medicine, Babol University of Medical Sciences, Babol, Iran

**Keywords:** Lateral epicondylitis, Tennis elbow, Injection therapies, Systematic review, Network meta-analysis

## Abstract

**Background:**

We aimed to compare the efficacy of local injection therapies for lateral epicondylitis in a Bayesian framework.

**Methods:**

We searched the Embase, PubMed, Cochrane Central Register of Controlled Trials, Web of Science, Scopus, and ProQuest, for randomized controlled trials published from inception to February 2021 in any languages. The injection therapies included corticosteroids (CSs), autologous blood (AB), botulinum toxin (BT), and platelet-rich plasma (PRP). Placebo was the reference group for comparison. The study outcomes were pain, function, and strength, at 1, 3 and 6 months after injection.

**Results:**

Thirty-one trials were finally included in this network meta-analysis, comprising 1,948 patients. In the first month of treatment, CS and BT were more efficacious than placebo in terms of pain reduction, and CS was superior to BT. In the same follow-up time, CS was also superior to placebo in terms of functional improvement. In the third month of treatment, BT was the only intervention that was more efficient than placebo in pain relief. With regard to functional improvement, none of the treatments significantly had a higher effectiveness than placebo in the same period. Moreover, no therapies were found to be more efficient than placebo in the sixth month of treatment in terms of any study outcomes. In addition, we did not identify an intervention superior to placebo regarding strength improvement outcome in any times of follow-up.

**Conclusion:**

CSs and BT are efficient in improving clinical outcomes of lateral epicondylitis in the short term. Also, the efficacy of CSs seems to be greater than BT. On the other hand, AB and PRP were not significantly more efficient than placebo in any times of follow-up.

Lateral epicondylitis, or tennis elbow, is a common cause of lateral elbow pain and can be seen in 1-3% of general population (1). It is stated that the problem is caused by a degenerative process due to repetitive microtrauma and strain along with vascular deprivation at the extensor tendon, typically extensor carpi radialis brevis tendon, which sometimes results in severe local pain that can impede proper upper limb function (2). There are various surgical and non-surgical treatments for lateral epicondylitis. However, it is recommended to select non-surgical treatments for the patients as much priority as possible, which include nonsteroidal anti-inflammatory drugs, electro physiotherapy, physical therapy, and local injection therapies. The usual injection therapies include corticosteroids (CSs), autologous blood (AB), botulinum toxin (BT), and platelet-rich plasma (PRP) (3, 4).

Until now, different studies have evaluated the effectiveness of injection therapies for lateral epicondylitis, but with contradictory findings (5, 6). In a meta-analysis study by Li et al. (7), it was shown that CSs were more effective than PRP in improving pain and function in a short-term (up to 2 months) follow-up, while in a long-term follow-up (6 months), the results were in favor of PRP. On the other hand, a network meta-analysis reported that CSs had small effect on improving pain compared to other injection therapies, and were not recommended (8). These conflicting results may be due to differences in the study objectives and methods. Previous meta-analyses had also some limitations, such as limited databases searched, or only English papers included. In the present study, we tried to comprehensively assessed the available evidence on the efficacy of local injection therapies for lateral epicondylitis with more precise objectives and overcoming the limitations of previous meta-analyses. These data will hopefully help clinicians to better manage the patients.

## Methods

**Study protocol: **The protocol of the present systematic review has previously been documented online in the PROSPERO registry (CRD42021244239). This study has been presented according to the guidelines of the Preferred Reporting Items for Systematic Review and Meta‐Analysis (PRISMA) extension statement for network meta-analyses (table S1) (9).

**Information sources and search strategy: **We performed a search on the literature published from inception to February 2021 in the bibliographic databases of the Embase, PubMed, Cochrane Central Register of Controlled Trials, Web of Science, Scopus, and ProQuest, without language restrictions. The relevant terms were searched in the Medical Subject Headings (MeSH) database, and finally, the keywords included “lateral epicondylitis” OR “tennis elbow” OR “lateral epicondylalgia” OR “elbow epicondylitis” AND “corticosteroid” OR “corticosteroids” OR “glucocorticoid” OR “glucocorticoids” OR “steroid” OR “steroids” OR “autologous blood” OR “botulinum toxin” OR “platelet-rich plasma” OR “PRP”. The search was limited to title or abstract. In addition, we conducted a hand search of the reference lists of relevant review articles and the retrieved papers for additional sources.

**Inclusion** **and exclusion criteria: **We included all randomized controlled trial (RCT) studies with the following criteria:

1. Including adult patients (aged ≥18 years).
2. Comparing clinical outcomes between at least two of the following treatments: CS, AB, BT, PRP, and placebo.
3. Investigating at least one of the following outcomes: visual analog score (VAS), disabilities of arm, shoulder and hand (DASH) score, modified Nirschl score (MNS), patient-related tennis elbow evaluation (PRTEE) score, grip strength (GS), at 1, 3, and/or 6 months.

The exclusion criteria were as follows:

1. Reviews, case reports, editorials, and letter to the editors.
2. Duplicate papers or evaluating the same sample.
3. Trials without clear methodology or results.
4. Full‐texts not being available

**Study selection and data extraction: **Two independent reviewers (MT, MZ) screened the titles and abstracts of all the articles obtained from the initial search for potential eligibility. Then, full-text of the potential papers were retrieved for final evaluation. Any disagreements related to the inclusion of articles were resolved by discussion between the investigators. The following data were extracted from each trial and finally entered into a Microsoft Excel spreadsheet (Microsoft Corporation, Redmond, Washington): first author’s name, study location (country), publication year, final follow-up (month), treatment names, total sample size, number of patients by gender (if available), patient’s age (if available), outcome measure. Non‐English papers were translated by Google Translate, where required. We contacted the corresponding authors by email if missing or unclear information existed. In case of duplicates, we selected those with the most comprehensive details.

**Risk of bias assessment: **Two authors (MT and MZ) independently contributed to the assessment of the quality of the included studies, using the revised Cochrane risk-of-bias tool for randomized trials (RoB 2) (10). This tool examines a study bias in five distinct domains, including randomization process, deviations from intended interventions, missing outcome data, outcome measurement, and selection of the reported result. Each domain has three quality levels, including ‘low risk’, ‘some concerns’, and ‘high risk’. 

**Study outcomes and statistical analysis: **The outcomes for the present network meta-analysis included:

1. “Pain intensity”, measuring by VAS (ranging from 0 [no pain] to 100 [worst pain] score, and MNS (ranging from 0 [no pain with exercise] to 4 [severe pain with normal activities] score).
2. “Function”, measuring by DASH (ranging from 0 [no disability] to 100 [most severe disability] score), and PRTEE (ranging from 0 [no disability] to 100 [significant disability] score).
3. “Strength”, measuring by GS (higher values mean more strength).

We did a network meta-analysis combining direct and indirect comparisons in a Bayesian framework using the R package ‘gemtc’ (https://cran.r-project.org/package=gemtc). With regard to the study outcomes, the pooled estimates were presented as the standardized mean difference (SMD) and 95% credible interval (CrI). SMD was selected as the effect size measure when the studies used different outcome scales, otherwise we used unstandardized mean difference (UMD). Data were combined using a random-effects model (11) to give more conservative estimates. Also, we used the Markov chains Monte Carlo method for all analyses. Node splitting models were used to obtain indirect estimates and to evaluate local inconsistency (12). Treatments were ranked for study outcomes using the surface under the cumulative ranking curve (SUCRA). We also presented the summary results of all pairwise comparisons and network meta-analysis in the league tables. For the present network meta-analysis, “placebo” was considered as the reference group for comparison. We assessed the publication bias with a comparison-adjusted funnel plot and Egger’s test. A p‐value <0.05 was considered significant for all relevant analyses.

## Results

**Fig 1:**
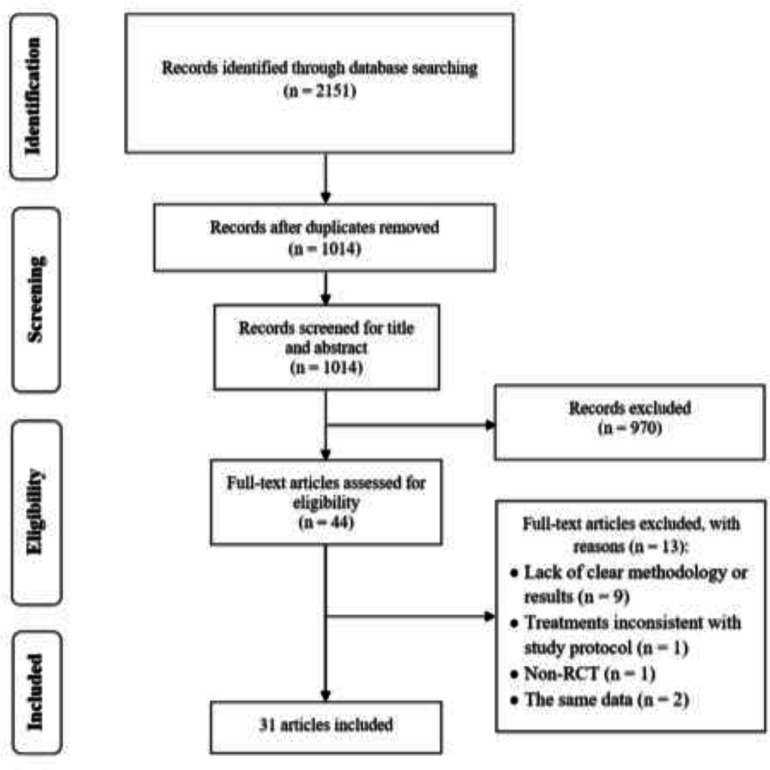
PRISMA flowdiagram

**Fig 2:**
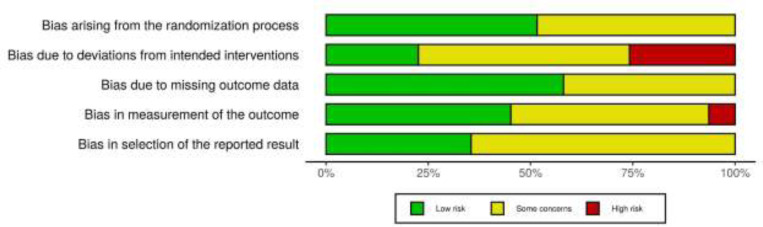
Risk of bias assessment for the individual domains

**Fig 3:**
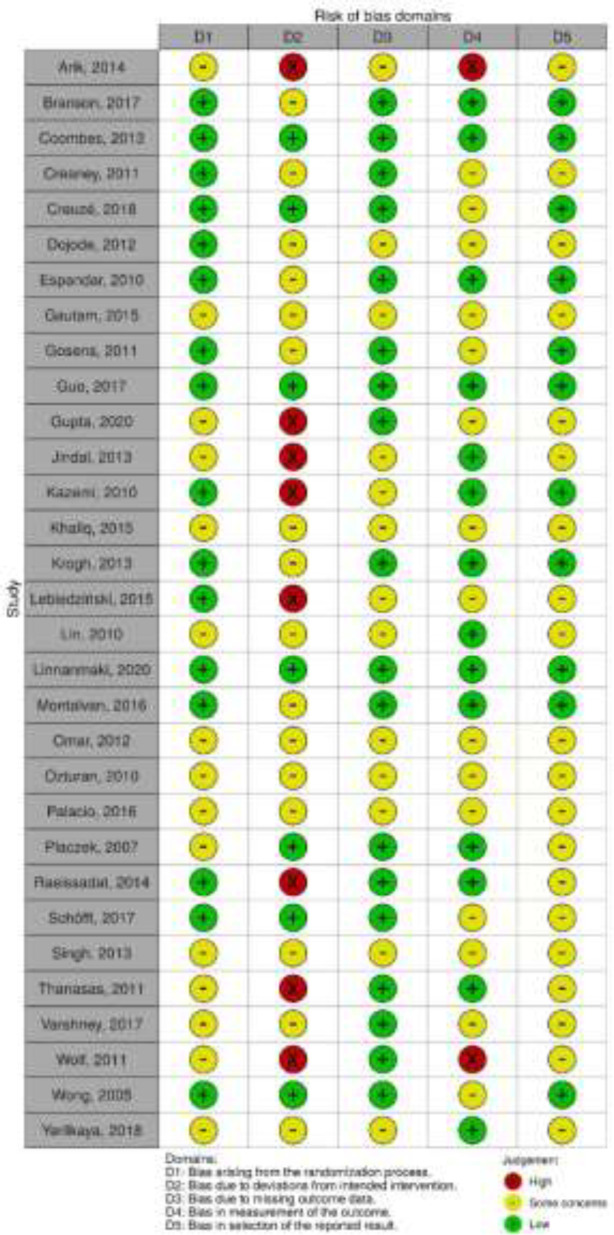
Risk of bias assessment for the individual studies

**Search results, study selection, and characteristics: **Searching the databases initially generated 2,151 records, of which 2,107 were excluded due to duplication or meeting exclusion criteria through screening titles or abstracts. Full-texts of 44 articles were evaluated, and finally, 31 eligible papers comprising 1948 patients with lateral epicondylitis were included in this systematic review (fig. 1). The pain intensity was investigated in 23 studies (13-35), the functional status was investigated in 16 studies (13, 14, 18, 20, 21, 23, 26, 28, 33, 36-42), and the strength was investigated in 8 studies (15, 17, 20, 21, 23, 25, 26, 43). Out of 23 trials assessing the pain reduction outcome, 20 studies used VAS pain score only, and 3 studies used both of VAS and MNS. Out of 16 RCTs investigating the functional improvement outcome, 8 studies used DASH only, 7 studies used PRTEE only, and one study used both. Baseline characteristics of the included RCTs were summarized in table 1. Moreover, the results of risk of bias assessment were reported for all of the included studies in figs. 2 and 3.

**Fig 4:**
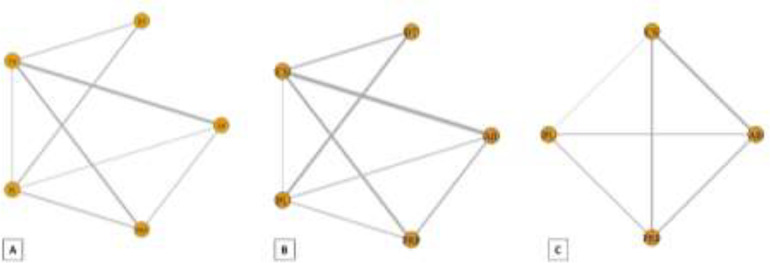
Network plot of comparisons for pain relief in the first (A), third (B) and sixth (C) month of treatment. Each node (circle) exhibits an injection therapy. The line width corresponds to the number of trials comparing the individual treatments

Abbreviations: CS, corticosteroid; AB, autologous blood; BT, botulinum toxin; PRP, platelet-rich plasma; PL, placebo

**Pain relief: **The results of pairwise and network meta-analyses about the pain relief outcome are represented in table 2. Due to different assessment tools, we used SMD to represent the pooled effect size. The network plots for the pain relief in different follow-ups have been provided in figs. 4A-C. The findings of inconsistency assessment and publication bias are also shown in figs. S1 and S2, respectively. Funnel plot and Egger’s test showed no publication bias in the network meta-analyses at any follow-ups. 

**Fig 5:**
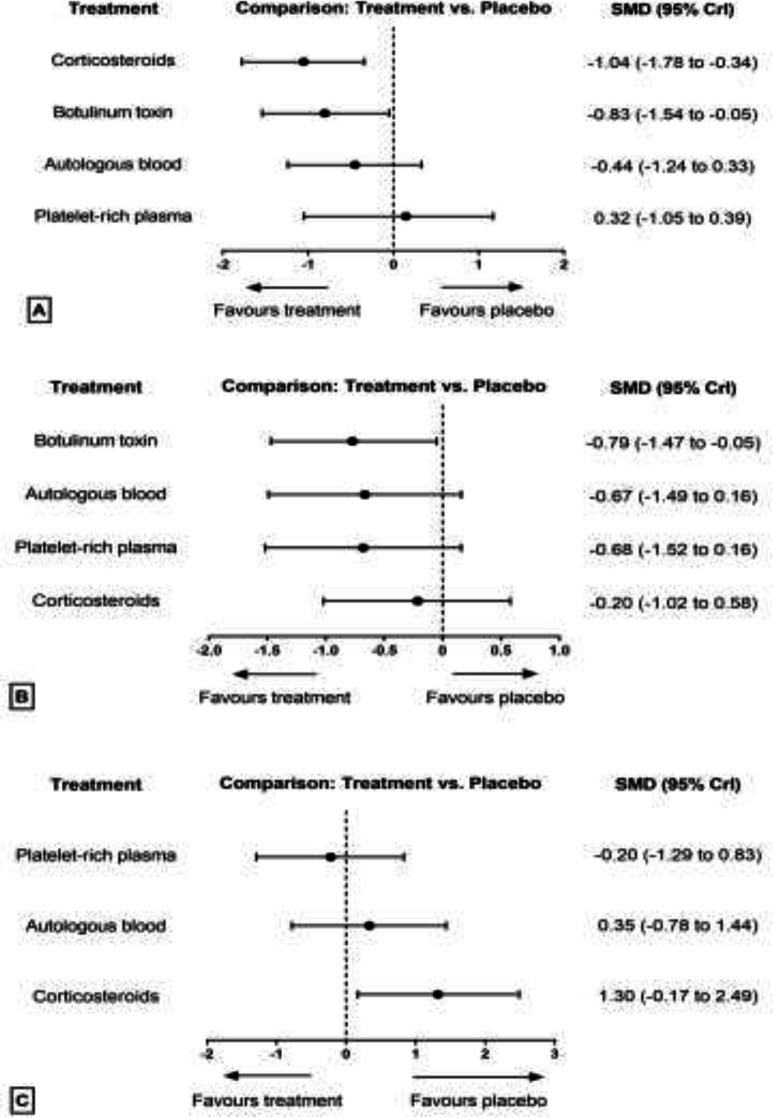
Forest plot of network meta-analysis results for pain relief in the first (A), third (B) and sixth (C) month of treatment. Treatments are ranked according to their SUCRA. Treatments crossing zero are not significantly different from placebo

Within the first month of follow-up, based on the network meta-analysis, CS had the most efficacy on the pain reduction versus placebo (SMD=-1.04, 95% CrI: -1.78 to -0.34), followed by BT (SMD=-0.83, 95% CrI: -1.54 to -0.05) (Fig 5A). Also, CS was ranked first and non-significantly more efficient than BT in pain relief. In the pairwise meta-analysis, BT was significantly associated with lower pain scores compared with placebo (SMD = -1.40, 95% CrI: -2.18 to -0.60).

Within the third month of follow-up, according to the network meta-analysis, BT was the only intervention that significantly decreased pain scores versus placebo (SMD = -0.79, 95% CrI: -1.47 to -0.05) (fig. 5B). Also, in the pairwise meta-analysis, BT was found to be superior to placebo (SMD = -1.30, CrI: -1.97 to -0.63).

Within sixth months follow-up, none of the treatments were significantly more efficient than placebo in pain reduction, either based on network meta-analysis or pairwise meta-analysis (fig. 5C).

**Fig 6:**
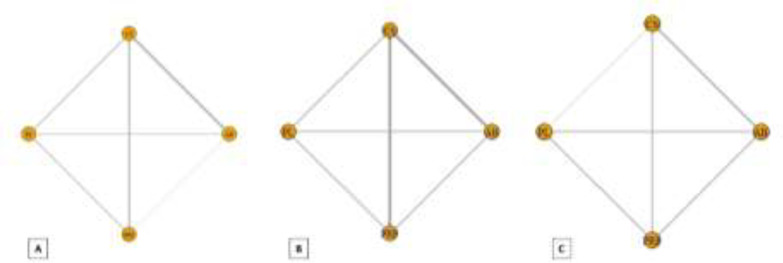
Network plot of comparisons for functional improvement in the first (A), third (B) and sixth (C) month of treatment. Each node (circle) exhibits an injection therapy. The line width corresponds to the number of trials comparing the individual treatments. Abbreviations: CS, corticosteroid; AB, autologous blood; PRP, platelet-rich plasma; PL, placebo

**Functional improvement: **The results of pairwise and network meta-analyses about the functional improvement outcome are represented in table 3. Due to different assessment tools, we used SMD to represent the pooled effect size. The network plots for the pain relief in different follow-ups have been provided in figs. 6A-C. The findings of inconsistency assessment and publication bias are also indicated in figs. S3 and S4, respectively. Funnel plot and Egger’s test showed no publication bias in the network meta-analyses at any follow-ups. 

**Fig 7:**
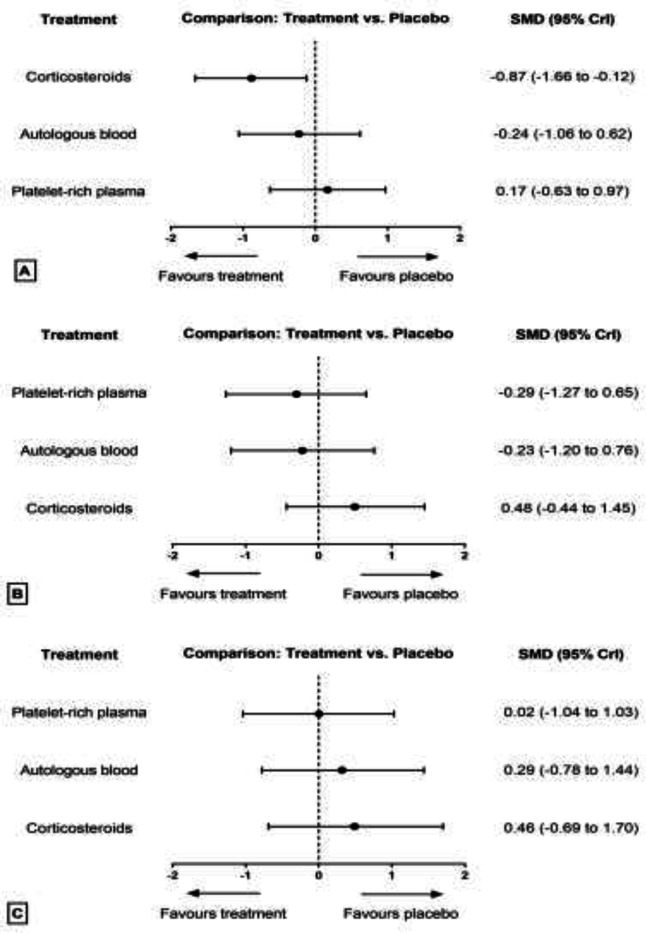
Forest plot of network meta-analysis results for functional improvement in the first (A), third (B) and sixth (C) month of treatment. Treatments are ranked according to their SUCRA. Treatments crossing zero are not significantly different from placebo

Within the first month of follow-up, network meta-analysis showed that CS was the only treatment that was significantly more effective than placebo in functional improvement (SMD = -0.87, 95% CrI: -1.66 to -0.12) (fig. 7A). However, no significant differences were seen between any treatments and placebo in functional improvement according to the pairwise meta-analysis.

Within the third and sixth months of follow-up, none of the injection therapies were significantly more efficient than placebo in functional improvement, either based on network meta-analysis or based on pairwise meta-analysis (figs. 7B-C). 

**Fig 8:**
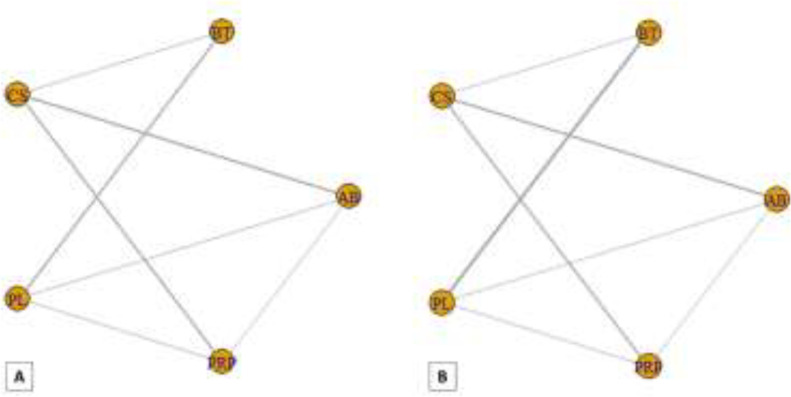
Network plot of comparisons for strength improvement in the first (A) and third (B) month of treatment. Each node (circle) exhibits an injection therapy. The line width corresponds to the number of trials comparing the individual treatments

Abbreviations: CS, corticosteroid; AB, autologous blood; BT, botulinum toxin; PRP, platelet-rich plasma; PL, placebo

**Strength improvement: **The results of pairwise and network meta-analyses about the strength improvement outcome are represented in table 4. Due to different assessment tools, we used SMD to represent the pooled effect size. The network plots for the pain relief in different follow-ups have been provided in figs. 8A-B. The findings of inconsistency assessment and publication bias are also shown in figs. S5 and S6, respectively. Funnel plot and Egger’s test showed no publication bias in the network meta-analyses at any follow-ups. 

**Fig 9:**
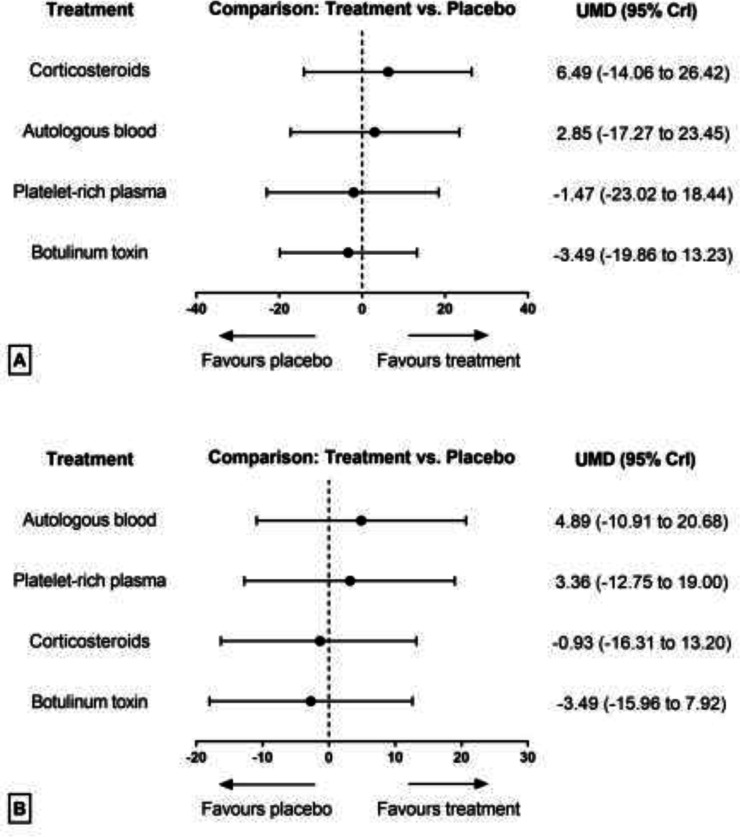
Forest plot of network meta-analysis results for functional improvement in the first (A) and third (B) month of treatment. Treatments are ranked according to their SUCRA. Treatments crossing zero are not significantly different from placebo

Within the first and third months of follow-up, none of the treatments were significantly more efficient than placebo in strength improvement, either based on network meta-analysis or based on pairwise meta-analysis (figs. 9A-B). Due to lack of enough data, performing network meta-analysis for strength improvement was not appliable in a sixth-month follow-up.

**Table 1:** Characteristics of the studies included in the systematic review and network meta-analysis

**First author**	**Publication year**	**Country**	**Final follow-up (m)**		**Outcomes**	**Treatment**	**Total patients (n)**	**Male (n)**	**Female (n)**	**Mean age (years)**
Arik (13)	2014	Turkey	6	VAS, PRTEE		AB	40	11	29	43.7
						CS	40	10	30	46.7
Branson (36)	2017	Australia	6	PRTEE		AB	14	10	4	47.9
						CS	14	8	6	48.1
Coombes (14)	2013	Australia	12	VAS, PRTEE		CS	43	27	16	49.3
						PL	41	14	17	49.9
Creaney (37)	2011	UK	6	PRTEE		PRP	63	36	27	53.0
						AB	48	27	21	48.0
Creuzé (15)	2018	France	3	VAS, GS		BT	30	17	13	47.3
						PL	30	16	14	46.7
Dojode (16)	2012	India	6	VAS, MNS		AB	30	13	17	42.9
						CS	30	12	18	42.2
Espandar (17)	2010	Iran	4	VAS, GS		BT	24	2	22	43.3
						PL	24	2	22	44.2
Gautam (18)	2015	India	6	VAS, DASH, GS		PRP	15	NA	NA	NA
						CS	15	NA	NA	NA
Gosens (19)	2011	Netherlands	24	VAS		PRP	51	23	28	46.8
						CS	49	23	26	47.3
Guo (20)	2017	Taiwan	4	VAS, PRTEE, GS		BT	15	6	9	49.9
						CS	11	5	6	53.4
Gupta (21)	2020	India	12	VAS, DASH, GS		PRP	40	NA	NA	42.4
						CS	40	NA	NA	39.4
Jindal (22)	2013	India	1	VAS, MNS		AB	25	14	11	39.0
						CS	25	17	8	37.3
Kazemi (23)	2010	Iran	2	VAS, MNS, DASH, GS		AB	30	7	23	47.2
						CS	30	4	26	47.0
Khaliq (24)	2015	Pakistan	1	VAS		PRP	51	21	30	34.0
						CS	51	24	27	34.0
Krogh (38)	2013	Denmark	3	PRTEE		PRP	20	9	11	47.6
						CS	20	11	9	44.7
						PL	20	9	11	43.9
Lebiedziński (39)	2015	Poland	12	DASH		AB	53	28	25	47.0
						CS	46	12	34	54.0
Lin (25)	2010	Taiwan	3	VAS, GS		BT	8	3	5	45.9
						CS	9	6	3	44.6
Linnanmaki (26)	2020	Finland	12	VAS, DASH, GS		PRP	40	18	22	46.0
						AB	40	20	20	46.0
						PL	39	17	22	49.0
Montalvan (27)	2016	France	12	VAS		PRP	25	17	8	47.0
						PL	25	17	8	46.4
Omar (28)	2012	Egypt	1.5	VAS, DASH		PRP	15	6	9	40.5
						CS	15	5	10	37.5
Ozturan (43)	2010	Turkey	12	GS		AB	18	7	11	44.0
						CS	20	10	10	45.8
Palacio (40)	2016	Brazil	6	PRTEE, DASH		PRP	20	NA	NA	46.6
						CS	20	NA	NA	46.2
Placzek (29)	2007	Germany	4	VAS		BT	68	31	37	47.4
						PL	62	30	32	46.9
Raeissadat (30)	2014	Iran	12	VAS		PRP	31	8	23	43.0
						AB	30	6	24	44.0
Schöffl (41)	2017	Germany	6	DASH		PRP	18	9	9	52.6
						PL	18	9	9	52.6
Singh (42)	2013	India	3	PRTEE		AB	30	12	18	35.2
						CS	30	16	14	33.0
Thanasas (31)	2011	Greece	6	VAS		PRP	14	NA	NA	35.9
						AB	14	NA	NA	36.6
Varshney (32)	2017	India	6	VAS		PRP	33	NA	NA	NA
						CS	50	NA	NA	NA
Wolf (33)	2011	USA	6	VAS, DASH		AB	9	NA	NA	NA
						CS	9	NA	NA	NA
						PL	10	NA	NA	NA
Wong (34)	2005	China	3	VAS		BT	30	5	25	45.0
						PL	30	6	24	44.2
Yerlikaya (35)	2018	Turkey	2	VAS		PRP	60	15	45	45.8
						PL	30	11	19	47.6

**Abbreviations:** VAS, visual analog score; DASH, Disabilities of Arm Shoulder and Hand score; MNS, modified Nirschl score; PRTEE, Patient-Related Tennis Elbow Evaluation score; GS, grip strength; CS, corticosteroid; AB, autologous blood; BT, botulinum toxin; PRP, platelet-rich plasma; PL, placebo

**Table 2:** Results of pairwise and network meta-analysis for pain relief in different follow-ups

**At 1** ^st^ ** month**	CS	-1.17 (-2.20 to -0.01)	-0.36 (-0.97 to 0.23)	-0.62 (-1.39 to 0.10)	-0.92 (-2.24 to 0.44)
	-0.22 (-1.06 to 0.59)	BT	NA	NA	-1.40 (-2.18 to -0.60)
	-0.60 (-1.14 to -0.04)	-0.37 (-1.32 to 0.58)	AB	0.27 (-0.75 to 1.34)	0.18 (-1.13 to 1.51)
	-0.70 (-0.13 to -1.28)	-0.48 (-1.38 to 0.42)	-0.11 (-0.79 to 0.56)	PRP	0.09 (-0.80 to 0.93)
	-1.04 (-1.78 to -0.34)	-0.83 (-1.54 to -0.05)	-0.44 (-1.24 to 0.33)	0.32 (-1.05 to 0.39)	PL
**At 3** ^rd^ ** month**	BT	NA	NA	0.27 (-0.65 to 1.17)	-1.30 (-1.97 to -0.63)
	-0.11 (0.99 to 0.82)	AB	0.31 (-0.65 to 1.29)	-0.73 (-1.37 to -0.08)	-0.04 (-1.22 to 1.19)
	-0.10 (-1.02 to 0.84)	0.01 (-0.68 to 0.69)	PRP	-0.52 (-1.34 to 0.33)	-0.03 (-1.12 to 1.13)
	-0.59 (-1.38 to 0.27)	-0.46 (-1.05 to 0.11)	-0.47 (-1.11 to 0.15)	CS	0.04 (-1.76 to 1.80)
	-0.79 (-1.47 to -0.05)	-0.67 (-1.49 to 0.16)	-0.68 (-1.52 to 0.16)	-0.20 (-1.02 to 0.58)	PL
**At 6** ^th^ ** month**	PRP	0.18 (-1.14 to 1.47)	-0.27 (-1.41 to 0.82)	-1.91 (-2.91 to -0.91)	
	-0.20 (-1.29 to 0.83)	PL	-0.13 (-1.45 to 1.07)	-0.48 (-2.26 to 1.34)	
	-0.54 (-1.36 to 0.26)	-0.35 (-1.44 to 0.78)	AB	-0.67 (-1.60 to 0.28)	
	-1.50 (-2.33 to -0.73)	-1.30 (-2.49 to -0.17)	-0.96 (-1.76 to -0.22)	CS	

**Note:** Treatments are ranked according to their SUCRA. The comparative therapeutic efficacies are reported as standardized mean difference with 95% credible intervals (in parentheses). The comparison between the treatments should be read from left to right. The network meta-analysis results are presented in the left lower (by comparing columns with rows), and the pairwise meta-analysis results are presented in the right upper (by comparing rows with columns). **Abbreviations:** CS, corticosteroid; AB, autologous blood; BT, botulinum toxin; PRP, platelet-rich plasma; PL, placebo

**Table 3.** Results of pairwise and network meta-analysis for functional improvement in different follow-ups

**At 1** ^st^ ** month**	CS	-0.52 (-1.18 to 0.14)	-0.94 (-2.02 to 0.30)	-1.11 (-2.04 to -0.24)
	-0.64 (-1.25 to -0.03)	AB	0.24 (-0.99 to 1.55)	-0.18 (-1.93 to 1.67)
	-0.87 (-1.66 to -0.12)	-0.24 (-1.06 to 0.62)	PL	-0.09 (-1.24 to 1.01)
	-1.04 (-1.74 to -0.37)	-0.41 (-1.21 to 0.41)	-0.17 (-0.97 to 0.63)	PRP
**At 3** ^rd^ ** month**	PRP	0.19 (-1.12 to 1.46)	-0.23 (-1.58 to 1.12)	0.84 (-1.71 to -0.02)
	-0.06 (-0.84 to 0.66)	AB	0.11 (-1.24 to 1.52)	-0.67 (-1.56 to 0.15)
	-0.29 (-1.27 to 0.65)	-0.23 (-1.20 to 0.76)	PL	-0.04 (-1.39 to 1.29)
	-0.77 (-1.44 to -0.16)	-0.71 (-1.36 to -0.08)	-0.48 (-1.45 to 0.44)	CS
**At 6** ^th^ ** month**	PRP	0.12 (-1.24 to 1.48)	0.38 (-0.04 to 0.91)	-1.13 (-1.76 to -0.63)
	0.02 (-1.04 to 1.03)	PL	-0.29 (-1.86 to 1.27)	-0.34 (-2.37 to 1.74)
	-0.27 (-1.23 to 0.63)	-0.29 (-1.44 to 0.78)	AB	0.42 (0.02 to 0.73)
	-0.44 (-1.39 to 0.48)	-0.46 (-1.70 to 0.69)	-0.16 (-1.02 to 0.68)	CS

**Note:** Treatments are ranked according to their SUCRA. The comparative therapeutic efficacies are reported as standardized mean difference with 95% credible intervals (in parentheses). The comparison between the treatments should be read from left to right. The network meta-analysis results are presented in the left lower (by comparing columns with rows), and the pairwise meta-analysis results are presented in the right upper (by comparing rows with columns).

**Abbreviations:** CS, corticosteroid; AB, autologous blood; PRP, platelet-rich plasma; PL, placebo

**Table 4:** Results of pairwise and network meta-analysis for strength improvement in different follow-ups

**At 1** ^st^ ** month**	CS	0.15 (-18.93 to 19.50)	NA	12.04 (-6.84 to 32.17)	9.37 (-18.44 to 38.14)
	3.67 (-12.57 to 19.27)	AB	0.07 (-30.72 to 31.67)	-2.39 (-33.30 to 28.09)	NA
	6.49 (-14.06 to 26.42)	2.85 (-17.27 to 23.45)	PL	-2.72 (-33.63 to 28.51)	3.50 (-16.99 to 23.32)
	7.81 (-7.10 to 24.68)	4.13 (-13.10 to 23.66)	1.47 (-18.44 to 23.02)	PRP	NA
	10.02 (-10.55 to 29.33)	6.70 (-15.79 to 26.87)	3.49 (-13.23 to 19.86)	2.21 (-21.30 to 22.57)	BT
**At 3** ^rd^ ** month**	AB	0.45 (-22.29 to 23.25)	2.86 (-20.62 to 26.96)	6.80 (-8.10 to 21.81)	NA
	1.46 (-12.01 to 15.06)	PRP	2.25 (-19.86 to 25.35)	4.28 (-10.58 to 19.90)	NA
	4.89 (-10.91 to 20.68)	3.36 (-12.75 to 19.00)	PL	NA	2.89 (-11.24 to 18.63)
	5.47 (-5.46 to 17.04)	4.06 (-6.89 to 15.86)	0.93 (-13.20 to 16.31)	CS	4.15 (-16.96 to 25.30)
	8.00 (-8.23 to 26.36)	6.62 (-9.30 to 23.54)	3.49 (-7.92 to 15.96)	2.79 (-12.60 to 18.02)	BT

**Note:** Treatments are ranked according to their SUCRA. The comparative therapeutic efficacies are reported as unstandardized mean difference with 95% credible intervals (in parentheses). The comparison between the treatments should be read from left to right. The network meta-analysis results are presented in the left lower (by comparing columns with rows), and the pairwise meta-analysis results are presented in the right upper (by comparing rows with columns).

**Abbreviations:** CS, corticosteroid; AB, autologous blood; BT, botulinum toxin; PRP, platelet-rich plasma; PL, placebo

## Discussion

In the present systematic review and network meta-analysis, we investigated the clinical efficacy of different injection therapies for lateral epicondylitis in different courses of follow-up. We assessed three different outcomes for this study, including pain reduction, functional improvement, and strength improvement. In the short-term follow-up (first month of treatment), it was found that CS and BT were more efficacious than placebo in terms of pain reduction, and CS was ranked first and was superior to BT. CS was also superior to placebo in terms of functional improvement in the short-term follow-up; However, we could not assess the efficacy of BT in this course due to insufficient data. In the mid-term follow-up (third month of treatment), BT was the only intervention that was more efficient than placebo in pain relief. Regarding functional improvement, none of the treatments significantly had a higher effectiveness than placebo in this period. Moreover, no therapies were found to be more efficient than placebo in the long-term follow-up (sixth month of treatment) in terms of any study outcomes. In addition, we did not identify an intervention superior to placebo regarding strength improvement outcome in any times of follow-up.

The anti-inflammatory mechanism of CSs can be explained by reduction of immune function and inflammatory cells and mediators, such as macrophages, mast cells, lymphocytes, prostaglandin and leukotrienes, leading to decrease in pain (44) Concerning BT, one mechanism can relate to temporary paralysis of the proximal extensors of the forearm, allowing a period of rest and aiding tissue recovery (45). In addition, releasing some mediators, such as calcitonin gene-related peptide, substance P, bradykinin, and glutamate, have been suggested as the other mechanism of analgesic properties of BT (45, 46).

So far, different meta-analyses have been conducted to explore the clinical effective of injection treatments for lateral epicondylitis. In the network meta-analysis by Dong et al. (8), hyaluronic acid and prolotherapy had significantly a higher efficacy than placebo in terms of pain reduction, and other treatments such as CS, PRP, AB and BT were not more efficacious compared with placebo. The present study has superiorities over Dong et al.’s study. First, we assessed the therapeutic efficacy of the injections at different times of follow-up, while Dong et al. did not categorize the follow-up times. Second, we evaluated three different outcomes in our study, whereas they investigated pain score only in their study. The other difference between the above-mentioned study and our network meta-analysis was the studied injection therapies, that is, we considered treatments that are more practical and have sufficient data for the analyses. There has been another network meta-analysis recently published related to the topic of our study (47); However, one of the major limitations of that study was lack of a placebo group for comparison. Also, we categorized the follow-up duration into three times, while that study grouped it into two periods. In the meta-analysis by Simental-Mendia et al. (48), the authors stated that PRP was not significantly more efficient than placebo in relieving pain and joint functionality, which was similar to our results. Other meta-analysis by Lin et al. (49) demonstrated that BT significantly reduced pain versus placebo within the first and third months of follow-up. They also declared that CS was more effective than placebo in the first month of treatment, but not in the next follow-ups. Additionally, they mentioned that CS is superior to BT in the first month of treatment. These results are similar to our findings obtained by pairwise or network meta-analyses.

In the present study, we attempted to overcome some of the limitations of the previous systematic review and meta-analyses by extending searched databases without language restriction, including placebo as the reference group for comparison, assessing three different clinical outcomes, and categorizing the follow-up duration into three different times (short-term, mid-term, and long-term).

This study has also some limitations. First, not enough data existed on follow-ups longer than six months to perform the network meta-analysis; however, considering that we did not find an efficient injection therapy in the sixth month, the presence of an efficient treatment in longer follow-ups might be improbable. Second, we witnessed wide and overlapped CrIs in some of the results, which were mainly due to limited number of studies. Therefore, it is needed to conduct more relevant RCTs. Third, the treatment substances and dosages sometimes differ between various trials and these factors need to be considered in further reviews when sufficient data are available for analysis.

In conclusion showed that corticosteroids and botulinum toxin are efficient in improving clinical outcomes of lateral epicondylitis. The effects of corticosteroids remained for one month. This time for botulinum toxin was three months. Also, the efficacy of corticosteroids seems to be greater than botulinum toxin within the first month of treatment. After three months, no significant therapeutic effects were found for corticosteroids or botulinum toxin. Regarding autologous blood and platelet-rich plasma, they were not significantly more efficient than placebo in any times of follow-up.

## Funding:

 None.

## Conflict of interests:

The authors declare no competing interests.

## Author contribution:

MT, RJ, SK and SME contributed to the study design. MT and MZ contributed to data collection. MZ contributed to data analysis. MT, RJ, SK and MZ contributed to drafting the manuscript. SME contributed to intellectual input and manuscript revision. All authors have read the manuscript and approved its final version.

## Data availability:

No additional data available.

## Ethics approval:

Not applicable.

## Consent to participate and for publication:

Not applicable.
